# Diagnostic Accuracy of Automated Pneumothorax Detection Via Novice-Acquired Ultrasound After Chest Tube Removal: Comparison With Expert Interpretation and Chest Radiography

**DOI:** 10.1016/j.chpulm.2026.100246

**Published:** 2026-03-10

**Authors:** Melissa Cote, Delaney Smith, Nicolas Orozco, Ben Huggard, Ben Wu, Khoa Tran, Benjamin Wilson, Niall Murphy, Blake VanBerlo, Robert Arntfield, Ross Prager

**Affiliations:** a Schulich School of Medicine, Western University, London, ON, Canada; b Deep Breathe Inc, London, ON, Canada; c Centro de Investigaciones Clínicas, Fundación Valle del Lili, Cali, Colombia; d Division of Critical Care Medicine, Western University, London, ON, Canada

**Keywords:** artificial intelligence, chest tube, diagnostic accuracy, lung ultrasound, novice users

## Abstract

**Background:**

Pneumothorax (PTX) is a frequent complication after chest tube removal, and timely detection is important to inform monitoring and potential intervention. Chest radiograph (CXR) remains the standard modality after chest-tube-removal PTX detection, despite its limited sensitivity and frequent delays in acquisition. Lung ultrasound (LUS) has superior accuracy and portability but is highly operator dependent, limiting its usability. The objective of this study is to evaluate whether artificial intelligence-assisted LUS (AI-LUS) enables novice users to accurately detect PTX after chest tube removal, compared with expert interpretation and CXR.

**Research Question:**

Does AI-LUS improve the ability of novice users to detect findings associated with PTX after chest tube removal compared with expert interpretation and CXR?

**Study Design and Methods:**

We conducted a prospective diagnostic accuracy study in adult patients undergoing chest tube removal at a tertiary academic hospital. LUS clips were acquired by novice operators using a handheld ultrasound device. A previously trained artificial intelligence model was then calibrated and used to detect the absence or presence of lung sliding. The reference standard was expert consensus LUS interpretation, with CXR serving as a secondary reference standard. Sensitivity and specificity were calculated at 2 time points: immediately after removal and after routine CXR.

**Results:**

A total of 76 patients were enrolled, yielding 848 LUS clips across 2 time points. Data from the first 12 patients were used to calibrate the model, with the remaining 64 forming the validation cohort. Compared with expert LUS interpretation, AI-LUS demonstrated a sensitivity of 0.775, a specificity of 0.831, and a negative predictive value of 0.96 for identifying absent lung sliding. When compared with CXR, AI-LUS achieved a sensitivity of 1.0 immediately after removal and 0.923 after CXR for PTX detection.

**Interpretation:**

Our results show that novice-performed AI-LUS demonstrated moderate diagnostic accuracy for detecting absent lung sliding after chest tube removal. Its very high sensitivity and excellent negative predictive value for identifying cases with absent lung sliding associated with PTX relative to CXR highlights a potential role for AI-LUS as a rapid triage tool that may reduce reliance on routine CXR, while acknowledging that PTX inference requires clinical correlation and additional ultrasound findings.

**Take-Home Points**

**Study** **Question:** Can artificial intelligence-assisted lung ultrasound (AI-LUS) enable novice users to reliably detect absent pleural lung sliding after chest tube removal compared with expert interpretation and chest radiograph (CXR)?

**Results:** Novice-performed AI-LUS demonstrated moderate agreement with expert lung ultrasound interpretation and high sensitivity with excellent negative predictive value for identifying cases associated with pneumothorax relative to CXR.

**Interpretation:** AI-LUS may serve as a rapid bedside triage tool after chest tube removal that could reduce reliance on routine CXR, while requiring appropriate clinical correlation and additional sonographic findings for pneumothorax diagnosis.

Chest tubes are commonly placed in patients recovering from cardiothoracic surgery or undergoing trauma resuscitation.^1^ As the patient recovers, chest tube management typically involves deactivating suction, clamping the tube, and ultimately removing it. At each step, reaccumulating pneumothorax (PTX) is routinely investigated and monitored to prevent the feared complication of tension PTX.^2^ Most commonly, screening is performed using chest radiograph (CXR) 1 to 8 hours after removal^3^; however, this practice is costly, time-consuming, and can delay disposition of patients.^3^^,^^4^

Lung ultrasound (LUS) is a safe, portable, rapid, and accurate bedside tool for diagnosing PTX.^5^ The absence of pleural lung sliding, while consistent with PTX, is nonspecific to the diagnosis, occurring in other conditions such as pleurodesis, pulmonary fibrosis, or hypoventilation.^6^^,^^7^ However, the identification of pleural lung sliding on LUS rules out PTX with near-perfect sensitivity at the site where the transducer is placed.^8^^,^^9^ This positions LUS as an ideal triage tool (high sensitivity) to rule out PTX across clinical contexts, including after chest tube removal. Although CXR has lower diagnostic performance than LUS for detecting PTX, the latter remains underused due to the need for specialized training and issues with interrater reliability.^10^, ^11^, ^12^ This makes a novice-useable, automated, and scalable solution to using LUS desirable.

Advancements in artificial intelligence (AI), particularly computer vision, have enabled the development of AI-assisted imaging tools for a range of clinical applications.^13^, ^14^, ^15^ AI interpretation of LUS has shown promise in detecting pleural lung sliding, but most studies remain retrospective, with limited data on real-time bedside performance.^16^, ^17^, ^18^ Retrospective evaluations report a sensitivity of 0.92 and a specificity of 0.89 for detecting absent lung sliding.^19^^,^^20^ In contrast, results from a prospective study with real-time deployment demonstrated slightly higher sensitivity (0.93) but reduced specificity (0.82), highlighting challenges in clinical translation.^21^^,^^22^

To our knowledge, no prospective studies have integrated novice-performed LUS into the diagnostic workflow. To our knowledge, this is the first study that considers incorporating novice-acquired LUS clips with retrospective AI support for PTX evaluation after chest tube removal. We evaluated AI interpretation of LUS clips collected by novice users at the bedside, focusing on diagnostic accuracy for absent lung sliding against multiple reference standards.

## Study Design and Methods

### Ethics Approval

This study was approved by the Western Research Ethics Board (Approval No. 119542). Informed consent was waived by the institutional review board due to the observational nature of the study, but all enrolled patients received a letter of information explaining the study and were provided contact details for withdrawal requests or questions.

### Study Design

We conducted a prospective diagnostic accuracy study to evaluate the performance of an AI-assisted LUS (AI-LUS) model in detecting the presence or absence of pleural lung sliding in hospitalized patients undergoing chest tube removal. LUS was performed at 2 time points: 5 to 30 minutes after removal, and several hours later during routine CXR. The study assessed the model’s ability to identify absent pleural lung sliding, a key sonographic sign of PTX, at time points before CXR findings are typically available.

### Setting

Patients were recruited from the cardiac-surgical ICU, trauma ICU, and thoracic surgery wards at a multisite tertiary academic hospital.

### Participants

We recruited adult patients (≥ 18 years of age) with a chest tube inserted for any indication that was scheduled for removal in the thoracic surgical ward or ICUs. Patients were excluded only if their clinical condition prevented LUS assessment at the time of examination. Patients were enrolled between July 2024 and August 2024. Recruitment occurred on 1 or 2 rotating weekdays per week, allowing for consecutive enrollment during each selected day. This strategy was designed to minimize spectrum bias and reduce the risk of inadvertently excluding potential confounders such as BMI and preexisting pulmonary conditions, all of which may influence the detection of pleural lung sliding.

### AI Algorithm Development

The AI algorithm used in this study was a binary classifier developed by Deep Breathe Inc, designed to detect the presence or absence of pleural lung sliding on brightness mode LUS. It was trained on 6,266 LUS clips from multiple clinical settings. Primary annotations of this development data set were assigned by a clinically trained reviewer. Challenging cases were flagged for secondary review by a certified critical care and emergency physician with > 20 years of LUS experience.

Before this study, the standalone performance of the algorithm was evaluated on an independent validation set of 818 clips. This validation set featured data from multiple vendors and diverse imaging conditions. Moreover, it was sourced from geographically distinct sites from those that formed the training set to ensure that performance estimates were unbiased and representative of real-world clinical variability. Labels were assigned based on consensus agreement of 3 board-certified US emergency physicians who were not involved in labeling the training set. On this validation set, the model achieved a sensitivity of 0.94 (95% CI, 0.90-0.97) and a specificity of 0.88 (95% CI, 0.85-0.90) for detecting absent pleural lung sliding.

For use in this study, the algorithm was recalibrated using clips from the 12 patients recruited before July 6, 2024, to optimize its performance under the study’s acquisition conditions (ultrasound probe vendor and preset: Butterfly IQ3 handheld ultrasound device equipped with a 1- to 10-MHz phased-array transducer using the abdominal preset).

### Novice Users

Novice users were defined as either medical students or nurses with no previous ultrasound training (eg, courses). This group included 2 medical student researchers and the patient’s bedside nurses. A study investigator provided only a standardized 5-minute bedside training session, including instructions on device operation, pleural window identification, and how to record a single clip once a stable image was obtained.

During each examination, patients were positioned in a sitting posture, and any dressings, ECG leads, or other barriers overlying the scanning area were temporarily removed to ensure unobstructed access for image acquisition.

### Index Test

The index test was the retrospective prediction of the AI-LUS algorithm on the novice-acquired LUS clips. All clips were acquired using a Butterfly IQ3 handheld ultrasound device equipped with a 1- to 10-MHz phased-array transducer using the abdominal preset. Depending on chest tube location, each participant’s anterior (R1/L1) and lateral (R2/L2) thoracic regions were screened. In total, 5 lung zones were recorded per time point: 4 lung zones at the anterior (R1/L1) sites and 1 lung zone at the lateral (R2/L2) sites. To evaluate absent lung sliding distribution and inform optimal probe placement, the right/left (R/L) zone was subdivided into 5 quadrants: upper medial, upper lateral, lower medial, lower lateral, and axillary (e-Fig 1).^22^ All R/L zones were systematically screened during each evaluation and only 1 clip per lung zone was permitted to prevent repeated testing that could artificially enhance performance. Index test users were not blinded to clinical information to reflect real-world use of AI-LUS within its intended clinical workflow.^23^

### Reference Standard

The primary reference standard (target condition of absent pleural lung sliding) was independent interpretation of the LUS images by 2 experts in LUS interpretation analyzing the same clips obtained by the novice user (the index test). Disagreements were resolved by a third expert who reviewed the clip and established a consensus interpretation. Of note, the reference standard readers were independent, blinded to clinical information, and blinded to the index test.

A secondary reference standard, the presence of any PTX of any size written in the CXR radiology report, was also applied for the target condition. This reference standard was used solely to evaluate the sensitivity of LUS because the superior diagnostic sensitivity of LUS compared with CXR would otherwise lead to an artificially low estimate of specificity.^5^^,^^11^^,^^12^

### Definition of and Rationale for Reference Test Positivity

Absent pleural lung sliding, the positive class, was defined as the absence of pleural shimmering at any point during the clip, regardless of location or duration. This definition captures lung point, identified as the boundary along the pleural line between 1 segment that exhibits pleural lung sliding and another that exhibits absent lung sliding. Present pleural lung sliding, the negative class, was defined as the continuous presence of pleural line motion throughout the clip. This definition captures lung pulse, a pleural movement caused by cardiac activity in the absence of respiration.^6^ Clips for which the presence or absence of pleural lung sliding could not be confidently determined due to factors such as poor image quality, motion artifacts, or unclear pleural line visualization were classified as indeterminate and excluded from the analysis. To reduce interobserver variability, all expert reviewers initially assessed a shared set of 20 pilot clips to establish interpretation criteria.

### Statistical Analysis

Categorical variables were reported as absolute counts and percentages. Normality of continuous variables was assessed using the Shapiro-Wilk test. Normally distributed variables were reported as mean with SD, whereas nonnormally distributed variables were reported as median with interquartile range (IQR). Sensitivity, specificity, positive predictive value, negative predictive value (NPV), and accuracy were then calculated. To assess the diagnostic performance of AI-LUS against CXR, ultrasound assessments performed at the postremoval and post-CXR time points were grouped by patient. If any of the regional assessments were positive for absent lung sliding, the overall evaluation was considered positive. Sensitivity was calculated using the CXR report as the reference standard for PTX. Subgroup analysis was performed considering the principal comorbidities of the cohort.

We performed a sample size calculation for a diagnostic accuracy study assuming the model’s previous sensitivity (0.94) and specificity (0.88), considering a prevalence of at least 1 clip with absent lung sliding per patient (38%), a desired power of 80%, and a 2-sided alpha of 5%; the estimated required sample size was 64 patients (the size of the validation cohort).

## Results

A total of 76 adult patients were enrolled in the study, including 12 in the calibration cohort and 64 in the validation cohort. The median age was 67.5 years (IQR, 59.0-74.0), and the median BMI was 27.9 kg/m^2^ (IQR, 23.6-31.9). The predominant indication for chest tube insertion was after surgery, representing 67 of patients (88.2%). Most participants were recruited from the cardiac-surgical ICU (n = 58, 76.3%). Common comorbidities in the validation cohort included obesity in 22 patients (34.4%), coronary artery disease in 13 patients (20.3%), myocardial infarction in 14 patients (21.9%), cancer in 7 patients (10.9%), congestive heart failure in 4 patients (6.2%), asthma in 5 patients (7.8%), and COPD in 4 patients (6.2%). Table 1 summarizes the patient demographics.

**Table 1:** Demographic and Clinical Characteristics of the Patients

Variable	Overall (N = 76)	Validation Cohort (n = 64)
Age, y	67.5 (59.0-74.0)	68.0 (59.0-73.2)
BMI, kg/m^2^	27.9 (23.6-31.9)	28.0 (23.9-31.9)
Missing	5	
Sex, male	51 (67.1)	42 (65.6)
Indication for chest tube		
Hemothorax/pneumothorax	6 (7.9)	6 (9.4)
Post surgical	70 (92.1)	58 (90.6)
Setting		
ICU	59 (77.6)	51 (79.7)
Medical ward	17 (22.4)	13 (20.3)
Comorbidities		
Obesity	25 (32.9)	22 (34.4)
Asthma	6 (7.9)	5 (7.8)
COPD	6 (7.9)	4 (6.2)
CKD	1 (1.3)	1 (1.6)
CAD	14 (18.4)	13 (20.3)
MI	17 (22.4)	14 (21.9)
Cancer	8 (10.5)	7 (10.9)
CHF	6 (7.9)	4 (6.2)
CXR findings		
Pneumothorax	16 (21.1)	13 (20.3)
Right side	5 (31.3)	3 (23.1)
Left side	9 (56.3)	8 (61.5)
Bilateral	2 (12.5)	2 (15.4)

Data are presented as No., No. (%), or median (IQR). CAD = coronary artery disease; CHF = congestive heart failure; CKD = chronic kidney disease; CXR = chest radiograph; IQR = interquartile range; MI = myocardial infarction.

A total of 848 LUS clips were collected from 64 patients at 2 time points: 386 clips after removal (n = 54), and 462 clips after CXR (n = 64). For analysis of AI-LUS diagnostic performance against expert interpretation, 768 clips from the postremoval and post-CXR time points were included, and 80 clips were excluded as indeterminate (29 clips from the postremoval and 51 from the post-CXR time points). The AI-LUS model demonstrated a sensitivity of 0.76, a specificity of 0.83, a positive predictive value of 0.40, and a NPV of 0.96 for detecting absent pleural lung sliding (Table 2).

**Table 2:** Comparison of AI-LUS and Expert Interpretation in the Detection of Absent Lung Sliding

AI-LUS Results		Expert Interpretation	
		Negative	Positive
AI-LUS prediction	Negative	557	22
	Positive	113	76

**Metrics**			
**Sensitivity (95% CI)**	**Specificity (95% CI)**	**PPV (95% CI)**	**NPV (95% CI)**
0.76 (0.68-0.85)	0.83 (0.80-0.86)	0.40 (0.33-0.48)	0.96 (0.94-0.98)

AI-LUS = artificial intelligence-assisted lung ultrasound; NPV = negative predictive value; PPV = positive predictive value.

In the validation cohort, 13 patients (20.3%) were diagnosed with PTX on postremoval CXR, including 8 left-sided, 3 right-sided, and 2 bilateral cases. When comparing AI-LUS predictions at the postremoval time point to the CXR reference standard, the model achieved a sensitivity of 1.00 for PTX detection. At the post-CXR time point, sensitivity was 0.93 (e-Table 1). In comparison, expert interpretation demonstrated a sensitivity of 0.92 at the postremoval time point and 0.69 at the post-CXR time point for PTX detection (e-Table 1).

**Figure 1:**
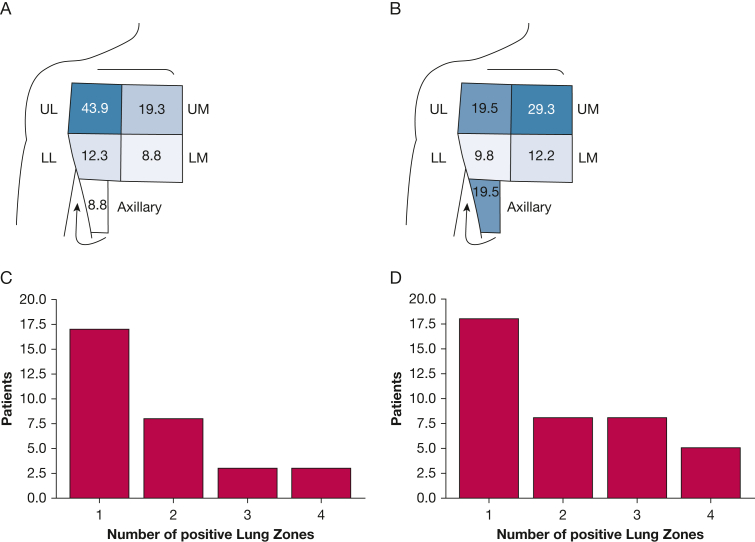
A-D, Distribution of expert interpretation of absent lung sliding at postremoval and post-CXR time points. Distribution of absent lung sliding at the postremoval time point (A) and the post-CXR time point (B). Patient distribution by number of lung zones with absent lung sliding at the postremoval (C) and post-CXR assessments (D). CXR = chest radiograph; LL = lower lateral; LM = lower medial; UL = upper lateral; UM = upper medial.

Figure 1 summarizes the anatomic distribution and frequency of lung zones with absent pleural lung sliding across the study cohort. Figures 1A and 1B present schematic heatmaps at the postremoval and post-CXR time points, respectively. At the postremoval time point (Fig 1A), absent sliding was most frequently localized to the upper lateral zone (43.9%), followed by the upper medial (19.3%), whereas the lower zones contributed less frequently (8.8%-12.3%). By contrast, at the post-CXR assessment (Fig 1B), the distribution shifted, with the upper medial zone representing the highest proportion of findings (29.3%) and notable involvement of both the upper lateral and axillary zones (19.5% each) (e-Table 2). Figures 1C and 1D illustrate the distribution of the number of positive lung zones (zones where absent lung sliding was observed), per patient. Most cases were limited to a single positive zone, whereas multizone involvement was less frequent, for both postremoval and post-CXR time points.

### Subgroup Analysis

Diagnostic performance of AI-LUS remained consistent across clinical subgroups. In patients with obesity, sensitivity was 0.80, specificity was 0.93, and NPV was 0.97, whereas those with chronic heart failure showed perfect sensitivity (1.00), a specificity of 0.87, and a NPV of 1.00. Patients with broader respiratory disease showed a sensitivity of 0.90, a specificity of 0.87, and a NPV of 0.98. More details are depicted in e-Table 3.

## Discussion

In this prospective study, we evaluated the diagnostic accuracy of novice-performed AI-LUS for the detection of absent pleural lung sliding and its utility in identifying PTX after chest tube removal. Although novice-performed AI-LUS demonstrated only moderate diagnostic accuracy for the identification of absent pleural lung sliding, it showed excellent sensitivity in ruling out PTX when compared with CXR. This supports the ability for novice-performed AI-LUS to serve as a screening (triage) test for PTX after chest tube removal.

Although AI-LUS showed only moderate sensitivity for detecting absent pleural lung sliding, its NPV remained high (approximately 0.96), indicating that a negative result reliably excluded the condition after chest tube removal. This finding is further supported by subgroup analyses, where performance metrics were stable across key clinical conditions such as obesity, COPD, asthma, and cancer (e-Table 2). The robustness of the NPV across these diverse patient groups and morbid population highlights the potential of novice-performed, AI-LUS to function as a safe and pragmatic triage tool in postcardiac surgery follow-up.

Compared with our previous retrospective and prospective work in which AI-LUS was performed on expert acquired images, the diagnostic accuracy of novice-performed AI-LUS was considerably lower, for which several factors likely contributed.^16^^,^^19^^,^^22^ First, novice users received only brief instruction (approximately 5 minutes), which may have limited image quality; as demonstrated by Singer et al,^24^ novice users may have difficulty producing images of comparable quality and achieving diagnostic performance equivalent to that of experts. Importantly, these findings highlight that image acquisition guidance is critical to ensure AI models receive diagnostically reliable input, especially in the context of novice sonographers. Additionally, novice users acquired images using the abdominal preset on the probe, whereas the AI model had been trained primarily on clips acquired using a lung preset, constituting a data set shift. To address this, the model was calibrated using a calibration data set; however, future models intended for deployment on devices in abdominal mode will likely require additional dedicated training.

When considering clinical application, the most important finding of this study was the high sensitivity of AI-LUS for PTX detection relative to CXR, with a sensitivity of 1.00 immediately after removal and 0.92 at the time of confirmatory CXR. These results suggest that LUS is as sensitive as CXR for screening PTX, while being faster to perform at the bedside and radiation-free; it therefore represents a viable alternative screening strategy in postcardiac surgery follow-up.^12^^,^^20^ This has meaningful health system implications because a negative LUS result immediately after chest tube removal could remove the need for confirmatory imaging, allowing earlier discharge from the ICU or hospital and reducing reliance on radiography.^22^ At our institution, replacing routine CXR with LUS for PTX screening has previously been estimated to reduce annual imaging costs from CAD $1.4 million to CAD $0.84 million, most of which reflects physician time rather than equipment costs.^4^ Wider use of AI-LUS and novice deployment could further increase these cost savings.

Although the overall sensitivity of AI-LUS relative to CXR was high, it is worth considering the single case in which AI-LUS missed a PTX identified on CXR at the post-CXR time point, resulting in a 0.93 sensitivity on 14 total cases. This occurred in a patient with 2 right-sided chest tubes, where only the apical tube was removed. Immediately after removal, AI-LUS correctly identified absent pleural lung sliding consistent with a small apical PTX. However, by the time of routine CXR, the presence of the remaining midlung tube may have altered the intrathoracic dynamics (either by partially evacuating air, limiting further air accumulation, or redistributing intrapleural gas). As a result, the PTX may have evolved in size or distribution such that it was no longer apparent on ultrasound, which could explain why it was not detected by AI at that time despite being observed on CXR.

Interestingly, in this study, AI-LUS demonstrated higher sensitivity for PTX detection than expert interpretation at both time points, even though all LUS images were acquired by novice operators. This finding may suggest that AI-assisted assessment can provide more consistent detection of subtle pleural abnormalities than human readers, potentially reducing variability in expert interpretation. Although this observation should be interpreted cautiously, it highlights the promise of AI-based LUS analysis as a reliable adjunct to expert clinical evaluation, particularly in postprocedural settings and when images are obtained by less experienced users.

As demonstrated in Figure 1, absent pleural lung sliding was distributed across multiple zones of the anterior and lateral chest, with no single zone consistently diagnostic. The superior chest was generally the most sensitive location immediately after removal, but absent lung sliding was not confined to this region. Reliance on a single zone would therefore miss some cases, highlighting the importance of multizone scanning protocols.^24^^,^^25^ These findings should guide future directives for novice training, emphasizing that although the upper zones are high yield, broader coverage is needed to avoid false-negative results. This consideration is particularly important in postcardiothoracic surgery patients, where adhesions or pleurodesis may produce loculated PTX that do not follow typical anterior distributions.

### Limitations

This study has several limitations. The absence of subgroup analyses (eg, patient positioning, ventilatory support) may limit generalizability. Importantly, our reference was substandard: primary outcome adjudication relied on multireader expert interpretation of the same LUS clips and CXR, rather than CT scans, which are considered the gold standard for diagnosing PTX. The lack of CT confirmation introduces potential misclassification, particularly for small or loculated PTXs. The indeterminate clips (clips with poor image quality, motion artifacts, or unclear pleural line visualization) were excluded, which may have slightly overestimated model performance by removing technically difficult acquisitions. Additionally, clustering or longitudinal correlations were not explicitly modeled due to unbalanced and incomplete repeated measurements across time points, which may have affected variance estimation. Also, image acquisition in this population may have been complicated by extensive instrumentation, dressings, and surgical changes, including ECG sensors and sternotomy scars. Finally, the cohort included predominantly postsurgical patients, and most of them were recruited from a cardiac-surgical ICU, representing a limitation to the generalizability and applicability of the results to non-postoperative settings (eg, trauma patients, spontaneous PTX).

### Future Directions

This preliminary research supports the feasibility of novice-performed AI-LUS to screen for PTX after thoracic intervention like chest tube removal. Future implementation of AI-LUS for novice users will require optimization of probe placement and acquisition guidance. Usability studies should be conducted to discover how the application workflow can be improved for novice sonographers. Additional fine-tuning with data collected by novices to optimize model performance is warranted, as is a more robust exploration of optimal novice guidance to improve diagnostic performance. Future studies incorporating CT validation, even in a targeted subset, would strengthen diagnostic benchmarking and external validity.

## Interpretation

Our results show that novice-performed AI-LUS to screen for absent lung sliding suggesting PTX after chest tube removal is feasible. AI-LUS, when performed by novices with minimal-to-no instruction, had moderate diagnostic accuracy for absent pleural lung sliding, and high sensitivity to diagnose PTX after chest tube removal, highlighting the potential role as a rapid triage tool. LUS-based screening for absent lung sliding after chest tube removal may be an alternative strategy instead of CXR, especially when additional advances in AI-LUS research support wide adoption by novice providers.

## Funding/Support

The authors have reported to *CHEST Pulmonary* that no funding was received for this study.

## Financial/Nonfinancial Disclosures

The authors have reported to *CHEST Pulmonary* the following: R. A. is the founder of Deep Breathe Inc, an AI ultrasound company that produced the model used in this study. R. P. is a consultant for Deep Breathe Inc. D. S., K. T., B. H., B. Wu, and B. V. are employees at Deep Breathe Inc. None declared (M. C., N. O., B. Wilson, N. M.).

## Supplementary Data

e-Online Data
