# Supplementary material for: Diagnostic Accuracy of Automated Pneumothorax Detection Via Novice-Acquired Ultrasound After Chest Tube Removal: Comparison With Expert Interpretation and Chest Radiography
Source: CHEST Pulm. 2026 Mar 10;4(2):100246. doi: 10.1016/j.chpulm.2026.100246 (PMC13419162; doi:10.1016/j.chpulm.2026.100246)
Supplement: e-Online Data [file mmc1.docx]

**Diagnostic Accuracy of AI-LUS for Pneumothorax Detection: Evaluating the Optimal Timing Post-Chest Tube Removal**

**AUTHORS AND AFFILIATIONS:**

Melissa Cote MSc^1^; Delaney Smith MMath^2^; Nicolas Orozco MD MSc^3^; Ben Huggard BSc^2^; Ben Wu BCS^2^; Khoa Tran MSc^2^; Benjamin Wilson RN^4^; Niall Murphy BSc^1^; Blake VanBerlo PhD^2^; Robert Arntfield MD^2,4^; Ross Prager MD^4^

1. Schulich School of Medicine, Western University, London, Ontario, Canada.
2. Deep Breathe Inc, London, Ontario, Canada.
3. Centro de Investigaciones Clínicas, Fundación Valle del Lili, Cali, Colombia.
4. Division of Critical Care Medicine, Western University, London, ON, Canada

**Supplemental Material**

**TABLE OF CONTENTS**

**e-Figure 1. Lung Ultrasound Zones Protocol 3**

**e-Table 1. Comparison of AI-LUS and Expert Interpretation versus Chest X-Ray at Post-removal and Post-CXR Time points 6**

**e-Table 2. Expert Interpretation Positive Lung Zones Distributions at Post-removal and Post-CXR Time points 4**

**e-Table 3. Subgroup Analysis of Diagnostic Accuracy of AI-LUS vs Expert 5**

**e-Figure 1: Lung Ultrasound Zones Protocol**


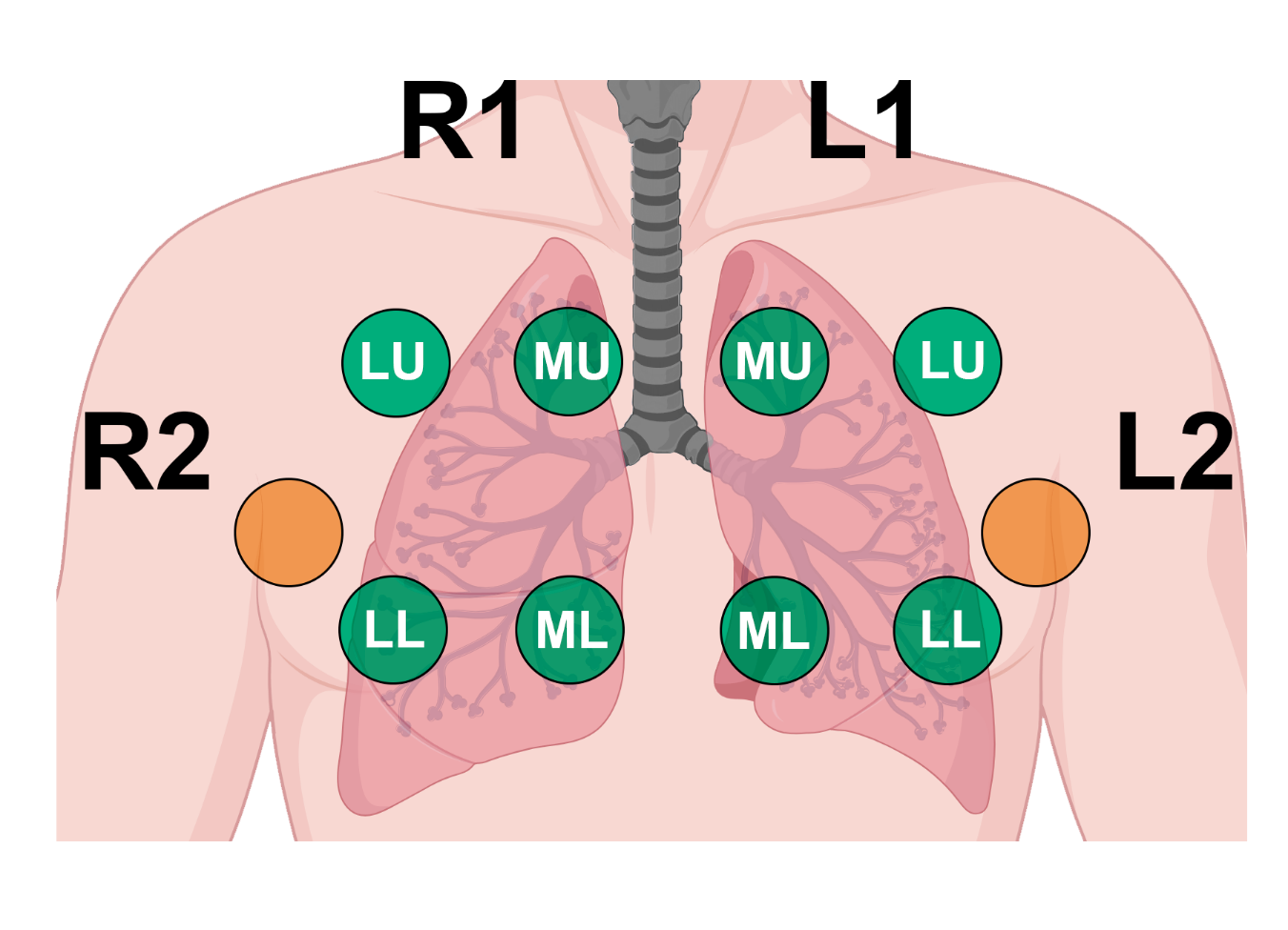


Abbreviations: *L* – Left side; *R* – Right side; *LU* – Lateral upper; *MU* – Medial upper; *LL* – Lateral lower; *ML* – Medial lower.

Green circles denote the anterior thoracic lung zones (R1/L1), and orange circles denote the axillary lung zones (R2/L2).

**e-Table 1. Comparison of AI-LUS and Expert Interpretation versus Chest X-Ray at Post-removal and Post-CXR Time points**

| **Sensitivity** | **AI-LUS (95% CI)** | **Expert (95% CI)** |
| --- | --- | --- |
| **Post Removal** | 1.00 (0.74, 1.00) | 0.92 (0.62, 1.00) |
| **Post CXR** | 0.92 (0.64, 1.00) | 0.69 (0.39, 0.91) |
| PTX detected by CXR (n= 13/64 patients) | | |

Abbreviations: *CXR* - Chest X-Ray

**e-Table 2. Expert Interpretation Positive Lung Zones Distributions at Post-removal and Post-CXR Time points**

| **Lung Zone** | **Time point** | **Count positive clips** | **Total Positive Clips per time point** | **Percentage** |
| --- | --- | --- | --- | --- |
| Upper Lateral | Post removal | 25 | 57 | 43.9% |
| Upper Medial |  | 11 |  | 19.3% |
| Lower Lateral |  | 7 |  | 12.3% |
| Lower Medial |  | 9 |  | 15.8% |
| Axillary |  | 5 |  | 8.8% |
| Indeterminate clips excluded |  | 29 | | |
| Upper Lateral | Post CXR | 4 | 41 | 9.8% |
| Upper Medial |  | 9 |  | 22.0% |
| Lower Lateral |  | 8 |  | 19.5% |
| Lower Medial |  | 12 |  | 29.3% |
| Axillary |  | 8 |  | 19.5% |
| Indeterminate clips excluded |  | 51 | | |

Abbreviations: *CXR* - Chest X-Ray

**e-Table 3. Subgroup Analysis of Diagnostic Accuracy of AI-LUS vs Expert**

| **Subgroup^a^** | | **Number of Patients** | **Number of Clips^b^** | **Sensitivity** | **Specificity** | **PPV** | **NPV** |
| --- | --- | --- | --- | --- | --- | --- | --- |
| **Obesity** | Yes | 22 | 306 | 0.80 | 0.88 | 0.49 | 0.97 |
|  | No | 42 | 860 | 0.76 | 0.81 | 0.37 | 0.96 |
| **COPD** | Yes | 4 | 56 | 0.75 | 0.93 | 0.60 | 0.96 |
|  | No | 60 | 1110 | 0.77 | 0.83 | 0.39 | 0.96 |
| **Asthma** | Yes | 5 | 57 | 1.00 | 0.82 | 0.50 | 1.00 |
|  | No | 59 | 1109 | 0.76 | 0.83 | 0.39 | 0.96 |
| **Cancer** | Yes | 7 | 76 | 0.67 | 0.93 | 0.57 | 0.95 |
|  | No | 57 | 1090 | 0.78 | 0.82 | 0.39 | 0.96 |
| **CHF** | Yes | 4 | 53 | 1.00 | 0.87 | 0.55 | 1.00 |
|  | No | 60 | 1113 | 0.76 | 0.83 | 0.39 | 0.96 |
| **Respiratory Disease** | Yes | 9 | 113 | 0.90 | 0.87 | 0.53 | 0.98 |
|  | No | 55 | 1053 | 0.76 | 0.83 | 0.37 | 0.96 |

Abbreviations: *PPV* – Positive Predictive Value; *NPV* – Negative Predictive Value; *COPD* – Chronic Obstructive Pulmonary Disease; *CHF* – Chronic Heart Failure.

^a^ Diagnostic performance of the subgroups was evaluated using the clips from the two timepoints (post removal and post-CXR). Reported metrics are calculated at the clip level.

^b^ A single patient could contribute as many as 10 clips across the two assessed time points; therefore, the aggregate number of clips does not directly correspond to the number of patients.
